# Combined transarterial chemoembolization and thermal ablation in candidates to liver transplantation with hepatocellular carcinoma: pathological findings and post-transplant outcome

**DOI:** 10.1007/s11547-024-01830-x

**Published:** 2024-06-03

**Authors:** Marco Fronda, Eleonora Susanna, Andrea Doriguzzi Breatta, Carlo Gazzera, Damiano Patrono, Federica Piccione, Luca Bertero, Fernanda Ciferri, Patrizia Carucci, Silvia Gaia, Emanuela Rolle, Giulia Vocino Trucco, Laura Bergamasco, Francesco Tandoi, Paola Cassoni, Renato Romagnoli, Paolo Fonio, Marco Calandri

**Affiliations:** 1 Department of Diagnostic Imaging and Interventional Radiology, City of Health and Science University Hospital of Turin, Turin, Italy; 2 Department of Surgical Sciences, University of Turin, Turin, Italy; 3 Liver Transplant Unit, General Surgery 2U, City of Health and Science University Hospital of Turin, Turin, Italy; 4 Pathology Unit, Department of Laboratory Medicine, City of Health and Science University Hospital of Turin, Turin, Italy; 5 Pathology Unit, Department of Medical Sciences, University of Turin, Turin, Italy; 6 Gastroenterology Unit, Department of Medical Sciences, City of Health and Science University Hospital of Turin, Turin, Italy; 7 University of Milan, Milano, Italy

**Keywords:** Hepatocellular carcinoma (HCC), Trans-catheter-arterial chemoembolization (TACE), Radiofrequency ablation, Microwave ablation, Liver transplantation, Pathologic response

## Abstract

**Objectives:**

Evaluating the pathological response and the survival outcomes of combined thermal ablation (TA) and transarterial chemoembolization (TACE) as a bridge or downstaging for liver transplantation (LT) in patients with hepatocellular carcinoma (HCC) > 3 cm.

**Materials and methods:**

A retrospective review encompassed 36 consecutive patients who underwent combined TA-TACE as bridging or downstaging before LT. Primary objectives included necrosis of the target lesion at explant pathology, post-LT overall survival (OS) and post-LT recurrence-free survival (RFS). For OS and RFS, a comparison with 170 patients subjected to TA alone for nodules <3 cm in size was also made.

**Results:**

Out of the 36 patients, 63.9% underwent TA-TACE as bridging, while 36.1% required downstaging. The average node size was 4.25 cm. All cases were discussed in a multidisciplinary tumor board to assess the best treatment for each patient. Half received radiofrequency (RF), and the other half underwent microwave (MW). All nodes underwent drug-eluting beads (DEB) TACE with epirubicin. The mean necrosis percentage was 65.9% in the RF+TACE group and 83.3% in the MW+TACE group (*p*-value = 0.099). OS was 100% at 1 year, 100% at 3 years and 94.7% at 5 years. RFS was 97.2% at 1 year, 94.4% at 3 years and 90% at 5 years. Despite the different sizes of the lesions, OS and RFS did not show significant differences with the cohort of patients subjected to TA alone.

**Conclusions:**

The study highlights the effectiveness of combined TA-TACE for HCC>3 cm, particularly for bridging and downstaging to LT, achieving OS and RFS rates significantly exceeding 80% at 1, 3 and 5 years.

## Background and aims of the study

Hepatocellular carcinoma (HCC) is the most frequent primary liver tumor, with more than 12.000 new cases diagnosed in Italy in 2022 [1]. Among the available treatments, liver transplantation (LT) is associated with the most favorable long-term survival [2, 3]. However, good overall (OS) and recurrence-free survival (RFS) after LT depend on the adhesion to strict selection criteria based on the size and number of HCC nodes and alpha-fetoprotein (AFP) level [4, 5]. In candidates to LT, locoregional therapies (LRT) are commonly employed to prevent dropout from the waiting list due to disease progression (bridging), or to reduce tumor burden in patients who do not meet selection criteria (downstaging). These therapies include thermal ablative (TA) therapies (radiofrequency or microwave ablation), trans-arterial chemoembolization (TACE), stereotactic ablative body radiotherapy (SABR) and selective internal radiation therapy (SIRT) [6]. A number of studies have demonstrated that effective downstaging is associated with good outcomes after LT [7, 8], with a net survival benefit compared to protracted LRT [9]. To enhance the possibility of achieving effective downstaging, some studies have suggested that, in HCC nodules > 3 cm in size, a combination of TA and TACE is more effective than TA or TACE alone as the synergistic effect allows the eradication of perilesional microsatellites that are frequently associated with lesions of this size [10–13]. However, although widely used in clinical practice, the combined TA-TACE therapy is not included in the main guidelines, likely due to the wide variability in its application across different centers, both in terms of patient selection and technique.

Thus, with the aim of quantifying the efficacy of a combined TA-TACE approach in the setting of LT, we conducted a retrospective study to assess the percentage of necrosis obtained with combined treatment of HCC nodules on livers explanted during LT, also evaluating the degree of concordance between radiological and pathological response. Moreover, we analyzed the overall survival (OS) and recurrence-free survival (RFS) after LT among patients who underwent TA-TACE treatment before LT for HCC > 3 cm and compared the survival outcomes of these patients with those treated by TA alone before LT for HCC < 3 cm.

## Materials and methods

### Study design and patient selection

This was a retrospective single-center study on adult (≥ 18-year-old) patients who underwent LT for HCC at our Institution between 2011 and 2021, focusing on those who had been treated by combined TA-TACE before LT. Inclusion criteria were: HCC diagnosed by pathologic assessment or non-invasive diagnosis criteria according to EASL-EORTC Clinical Practice Guidelines, LT between 2011 and 2021, therapeutic indication discussed at multidisciplinary tumor board, percutaneous combined TA + TACE performed as a bridging or downstaging before LT, target node diameter ≥ 3 cm and Eastern Cooperative Oncology Group (ECOG) performance status 0. Exclusion criteria were: target lesion size > 7 cm, anatomical location unsuitable for TA, advanced-stage HCC according to the BCLC staging system and Child–Pugh score > B7. Relevant clinical data, including patient and HCC characteristics at diagnosis and at LT time, imaging studies and details about LRT performed before LT were retrieved from our prospectively maintained institutional database and from clinical records. The time interval between TA and TACE and the order in which they were performed were assessed for each patient. The primary endpoint was pathological response, evaluated as the degree of necrosis of the target lesion on the explanted liver. Pathological response was also compared to radiological response on pre-LT imaging. Secondary endpoints were OS and RFS. Risk factors associated with HCC recurrence after LT were evaluated. Finally, to assess the yield of combined TA-TACE, patients treated by this approach were compared to a selected group of patients treated by TA alone with comparable follow-up period.

### Interventional procedures

The choice for a particular LRT was mainly guided by the size and location of the HCC node, with a combined approach being generally preferred for tumors ≥ 3 cm in diameter. In all cases, the therapeutic indication was determined during multidisciplinary tumor board meetings.

Thermal ablations were carried out, under intravenous conscious sedation, using either radiofrequency (RF) or microwave (MW), employing a percutaneous approach guided by ultrasound, CT, or a combination thereof, based on optimal tumor visibility. RF ablations were performed using the multi-needle LeVeen electrode (Boston Scientific), while for MW ablations the Emprint system with Thermosphere technology (Medtronic) was used.

**Fig. 1:**
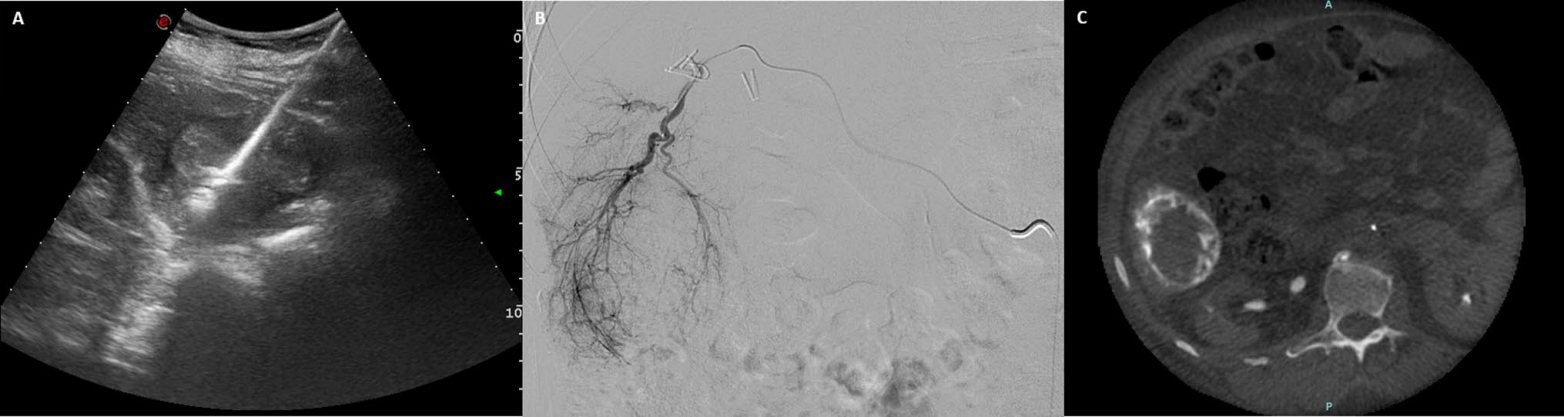
**a**–**c** Combined TA-TACE treatment. a. Microwave ablation of a 55-mm HCC nodule of the VI liver segment performed using a 14G probe (maximum power output 150 W, ablation time 3 min and 30 s), **b** Digital Subtraction Angiography (DSA) performed 24h after TA, showing the ablation zone surrounded by an hyper-vascular rim, **c** unenhanced cone beam CT performed at the end of chemoembolization, demonstrating the complete peripheral coverage of the ablation zone

Drug-eluting bead transarterial chemoembolization (DEB-TACE) was performed under local anesthesia, with femoral artery access established using a 5 French (F) sheath. The celiac trunk and, if deemed necessary based on pre-procedural CT images, the superior mesenteric artery, were catheterized using a 5 F catheter (Shepherd Hook II, TEMPO SHK1.0, Cordis). An angiography of the celiac trunk and/or superior mesenteric artery was conducted with image subtraction achieved by injecting 20 mL of iopromide (Ultravist 370, Bayer Pharma, Berlin, Germany) at a rate of 4 mL/s. Subsequently, segmental or subsegmental arteries supplying the hypervascular lesion were catheterized using a 1.9–2.7 F microcatheter (Progreat, Terumo—Progreat Lambda, Terumo—Carnelian, Tokai Medical Product) with 0.014-in or 0.016-in guidewire (Fathom; Boston Scientifics). Embolization was performed using drug eluting microspheres (DCBeads 100–300, Biocompatibles – Lifepearl 100 ± 25, Terumo), preloaded with 50 mg of epirubicin (Farmorubicin, Pfizer) diluted in a mixture of 15 mL saline solution and contrast medium (in a 50:50 ratio) and administered under fluoroscopic guidance to prevent reflux. The dosage administered was customized for each patient to achieve complete devascularization of the target lesion and stasis of the feeding vessels. If necessary, embolization was completed using non-loadable microparticles (Hydropearl 400 ± 75 µm, Terumo) until nearly complete flow stasis was achieved within the target vessel. For each patient, the time interval between the two procedures and their sequence (whether TA or TACE was performed first) were evaluated. An example case of combined treatment performed in our center is shown in Fig. 1.

### Pathological evaluation of response

**Fig. 2:**
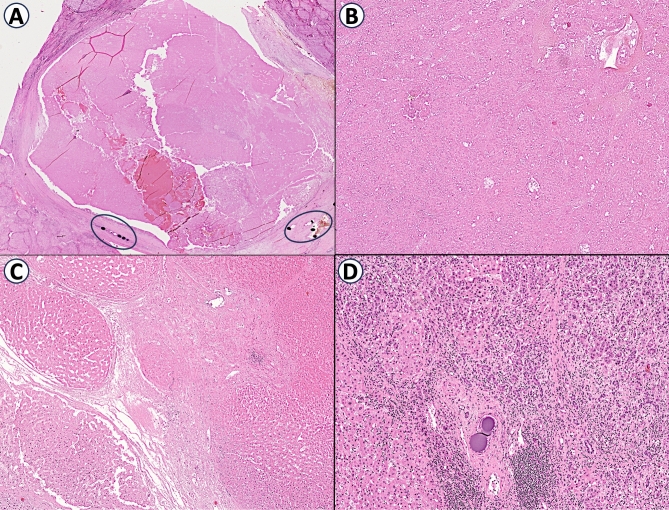
Histopathological features of postoperative tumor nodules. Most of the tumor nodules exhibited expansive margins of growth and abundant necrosis. Additionally, clusters of chemoembolization substance within blood vessels were frequently noticed adjacent to these nodules, as highlighted in the black circles (**a** HE 2.5X). Tumor growth pattern varied, ranging from solid (**b** HE 10X) to trabecular (**c** HE 10X), but a combination of these two patterns was generally observed. From a cytological perspective, the neoplastic cells typically displayed discrete nuclear irregularities and prominent nucleoli, which are features consistent with an Edmondson-Steiner Grade G2. Moreover, the presence of a chronic inflammatory infiltrate was also frequently observed (**d** HE 20X)

For each node treated by combined therapy, an explant pathology review on formalin-fixed paraffin-embedded (FFPE) specimens was conducted. Specifically, the following aspects were assessed: the percentage of necrosis in the treated nodules, the pattern of tumor growth, the number of mitoses per 10 high-power fields (HPF), the type of margins, the grade according to modified Edmondson-Steiner criteria and the presence of lymphovascular invasion. The anatomopathological response was considered complete only when the achieved necrosis percentage was evaluated as 100%. An example of postoperative specimens is depicted in Fig. 2.

### Definitions

Radiological response was evaluated according to the modified response evaluation criteria in solid tumors (mRECIST) [14], through four-phase cross-sectional imaging (CT or MR) performed at least one month after the procedure. The examinations were evaluated by two independent radiologists, and disagreements in interpretation were resolved by discussion until consensus was reached. Model for end-stage liver disease (MELD) and albumin-bilirubin (ALBI) scores was calculated according to formulae in the original publications [15, 16]. Overall survival was defined as the time interval between the date of liver transplantation and the date of death or last follow-up, whereas RFS was defined as the time interval between the date of transplantation and the date of HCC recurrence. The post-LT follow-up was conducted using CT scans with personalized timing based on the RETREAT score [17]. Patient deaths without recurrence were treated as censors.

### Comparison with thermal ablation

In order to assess oncological outcomes of combined therapy, patients treated by TA-TACE were compared to a group of consecutive patients with HCC nodules ≤ 3 cm in size, treated by thermal ablation only, with comparable duration of the follow-up.

### Statistical analysis

Categorical values are expressed as percentages. Median values, along with their respective ranges, are used for continuous variables. Variables with a normal distribution are presented as mean ± standard deviation. Variables identified by univariate analysis as potential predictors of the event were included in two multivariate regressions: binary logistic regression (BLR), providing outcome p-values and odds ratios (OR) with 95% confidence intervals (CI), and Cox proportional-hazard regression, which assesses the impact of variables not only in terms of occurrence but also in the timing of the event, providing outcome p-values and risk ratios (RR) with 95% CI. The impact of a predictor on overall and recurrence-free survival was studied using Kaplan–Meier curves, and the comparison between two groups with different predictor values was made using the log-rank test. Hazard ratios (HR) with 95% CI were used to express the risk. The events per person-year rate (EPPY rate) was used to compare the event rate between two groups with different exposure times. The two compared groups were those subjected to combined therapy and those subjected to ablation alone. The statistical significance of the difference between two EPPY rates is expressed by the rate ratio (RR) and the p-value provided by the Z-test. Best subsets regression was utilized to highlight potential predictors of post-transplant recurrence. Factors with p-values < 0.05 and 95% CI for OR, RR and HR excluding the value 1 were considered statistically significant. All statistical analyses were performed using StatPlus for Macintosh Build 8.0.1.0/Core v7.7.11, 2021 (AnalystSoft, Walnut).

## Results

**Fig. 3:**
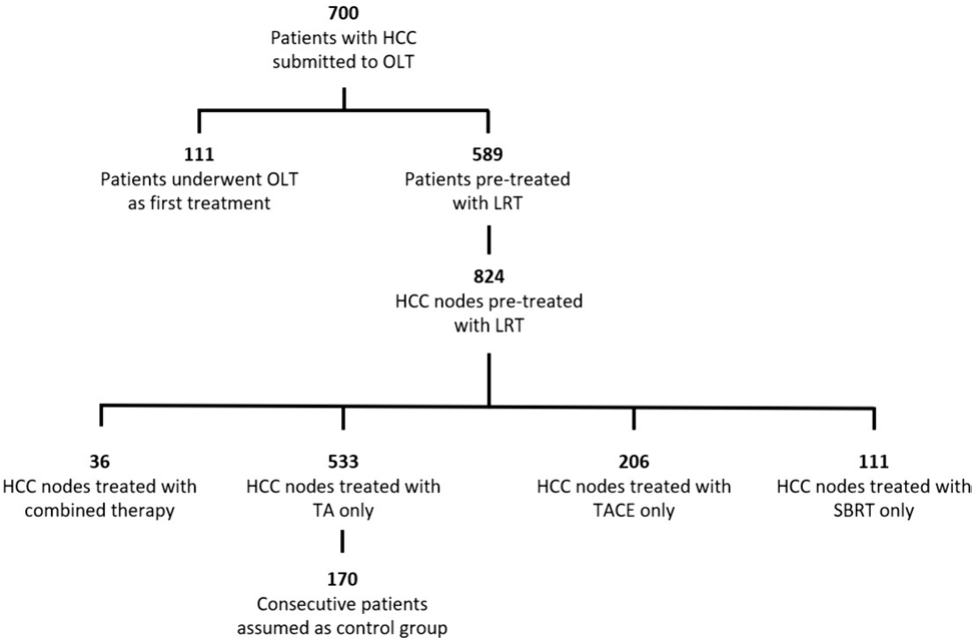
Flow diagram of patients’ selection. HCC Hepatocellular Carcinoma; Liver Transplant; LRT Locoregional Treatments; TACE Transarterial Chemoembolization

Seven-hundred patients with HCC undergoing LT were identified from our prospectively maintained institutional database. A hundred and eleven (15.8%) were excluded as no LRT was performed before LT. For the remaining 589 patients, a lesion-specific database was created, treating every HCC node as an entry. Overall, 824 HCC nodes were included, of which 533 (64.7%) were treated by thermal ablation alone, 206 (25%) by TACE, 49 (5.9%) by SBRT, and 36 (4.4%) were subjected to combined TA-TACE approach. As only one HCC node was treated by combined TA-TACE in each patient, the 36 nodes treated by a combined approach corresponded to 36 patients (Fig. 3).

Among the 36 patients included in the present study, 33 were males (91.7%) and 3 were females (8.3%); the median age was 64.9 years (range 52–72 years). The characteristics of the study population are summarized in Table 1. Eighteen (50%) patients had monofocal HCC at diagnosis; for the patients with multifocal HCC, treatments other than combined therapy were allowed for other lesions to meet criteria for LT. Twenty-three (63.9%) patients underwent combined therapy as a bridging to LT, while 13 (36.1%) were downstaged in order to access LT. The mean size of the nodes was 4.25 cm (range 3–6.8 cm). Out of these, 18 (50%) underwent RF treatment, while the remaining 18 (50%) underwent MW. With regard to chemoembolization treatment, all nodes underwent DEB-TACE. In 22 (61.1%) cases, ablation was performed first, whereas in 14 (38.9%) after TACE. The average number of days between the two procedures was 3.4 days (range 0–16 days). The median number of days between combined treatment and transplantation was 213 days (7.0 months). When stratifying these data based on tumor load, median waiting time was 216 days (7.1 months) in the bridging group and 210 days (6.9 months) in the downstaging group. Median follow-up after LT was 42.4 months.

**Table 1:** Study population: baseline characteristics, technical details and response rates

	Combined *N* = 36	Ablation only *N* = 170	*p*-value
*Gender*			
Male	33 (91.7%)	141 (82.9%)	0.3089
Female	3 (8.3%)	29 (17%)	
*Tumor presentation*			
Monofocal	18 (50%)	108 (63.5%)	0.1371
Multifocal	18 (50%)	62 (36.5%)	
*Target node size*			
Medium size (cm)	4.25	2.28	** < 0.0001**
*AFP*			
Last AFP level before LT (ng/mL)	13.87	28.24	0.1825
*Ablation method*			
RF	18 (50%)	116 (68.2)	0.053
MW	18 (50%)	54 (31.8)	
*Bridging versus Downstaging*			
Bridging	23 (63.9%)	134 (78.8%)	0.082
Downstaging	13 (36.1%)	36 (21.2)	
*Treatments sequence*			
Ablation first	22 (61.1%)		
TACE first	14 (38.9%)		
*Pathological response*			
Complete	13 (36.1%)		
Partial	23 (63.9%)		
*Radiological response*	*N* = 31	*N* = 154	
Complete	25 (80.6%)	131 (85%)	0.588
Partial	6 (19.4%)	23 (14.9%)	
Not available	5	16	

*RF* Radiofrequency, *MW* Microwave, *TACE* Transarterial chemoembolization

### Histopathological response

In 13 (36.1%) cases explant pathology revealed complete necrosis of the lesion, while the median extent of necrosis was 90%. When stratifying based on ablative methods, the RF + TACE group had 22% (4/18) complete anatomopathological responses and the MW + TACE group had 50% (9/18) complete responses (*p* = 0.289). Overall, the mean percentage of necrosis in the RF + TACE group was 65.9%, compared to 83.3% in the MW + TACE group (*p* = 0.099). The post-transplant anatomopathological analysis of the explanted liver also assessed HCC differentiation according to the modified Edmondson-Steiner grades. Out of the 22 nodes for which grading was assessable, 1 node was Grade G1 (4.5%), 16 nodes were Grade G2 (72.2%), and 5 nodes were Grade G3 (22.7%). For the 14 nodes with a necrosis percentage ≥ 95%, histological grading could not be evaluated. Potential predictors of complete anatomopathological response were investigated through univariate analysis. Among factors related to patient clinical characteristics, lesion characteristics and the technique used, none of the continuous or dichotomized variables significantly predicted complete histopathological response (Table 2).

**Table 2:** Univariate logistic regression for pathological complete response

	Complete AP response (*N* = 13)	Partial AP response (*N* = 23)	*P* value
Age	59 ± 5	60 ± 4	0.35
Central segment (S1, S4, S5 or S8)	54%	48%	> 0.99
Node diameter	4.3 ± 1	4.2 ± 0.7	0.83
Multifocal HCC	62%	43%	0.49
MW ablation	69.2%	52%	0.16
TACE as first procedure	58%	30%	0.13
Days between the two procedures	3.6 ± 4.9	3.3 ± 3.2	0.84
Downstaging	38%	35%	0.83
Pre-OLT AFP level	6.7 ± 7.4	17.2 ± 33.8	0.35
Pre-OLT Child–Pugh score	5.8 ± 1.6	5.4 ± 0.7	0.35
Pre-OLT MELD score	9.5 ± 2	9.3 ± 2.6	0.83
pre-OLT ALBI score	1.7 ± 0.7	1.4 ± 0.6	0.35

*HCC* Hepatocellular carcinoma, *MW* Microwave, *TACE* Transarterial chemoembolization, *AFP* Alpha fetoprotein, *MELD* model for end-stage liver disease, *ALBI* Albumin-bilirubin score

### Radiological response

**Fig. 4:**
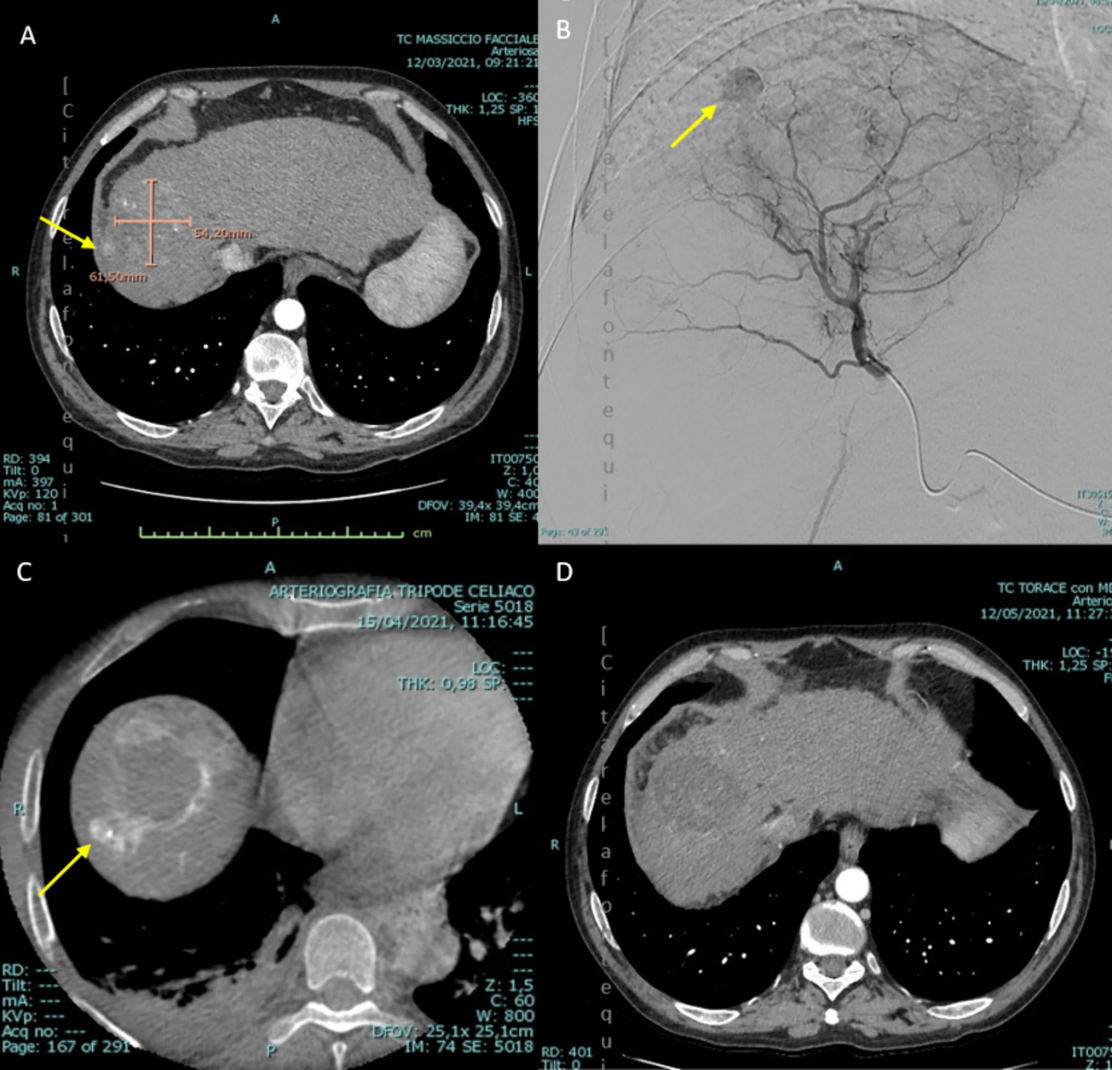
**a**–**d** Example case of successful combined treatment. a. The pre-treatment CT shows an HCC nodule (61,5 × 54 mm) with a satellite (arrow), **b** pre-TACE angiography showing the main node as characterized by a necrotic core and a peripheral enhancement; the satellite is still hyper-vascular, **c** cbCT performed immediately after TACE shows the complete enhancement of both the periphery of the ablation zone and the satellite, d. the CT performed one month after the combined treatment shows complete radiological response according to mRECIST criteria. At the same time, the patient’s AFP levels dropped from 15.867 to 3.2 ng/ml

Post-treatment four-phase CT or MR scans were available for 31 (86.1%) cases, as 5 patients underwent LT before the first planned radiological follow-up. In this group, the rate of radiological complete response (CR) was 80.6% (25/31) and the rate of partial responses (PR) was 19.4% (6/31), resulting in an overall objective response (OR) rate of 100%. An example of combined treatment leading to radiological CR is shown in Fig. 4. When assessing potential predictors of complete radiological response, only MW ablation showed a borderline significant association (*p* = 0.09). We analyzed the concordance between complete histopathological response (AP) and complete radiological response, obtaining an index of agreement (IoA) of 58%. The Cohen's kappa coefficient was *k* = 0.59 (CI 0.31–0.88), indicating moderate agreement. In particular, in 16 out of 31 cases (51.6%), the complete radiological response at the first follow-up was not confirmed as a complete response by the anatomopathological analysis. On the other hand, in 2 out of 31 cases (6.5%), complete response was not achieved at the first radiological follow-up, but the necrosis was deemed complete in the subsequent histopathological analysis. No significantly predictive variables of agreement between radiological and anatomopathological responses were identified.

### Overall and recurrence-free survival

Overall survival (OS) was 100% (36/36) at 1 year, 100% (36/36) at 3 years and 94.7% (18/19) at 5 years. In patients treated with combined therapy, survival after transplantation was stratified based on inclusion in the transplant criteria at diagnosis, which determined whether the treatment was performed as bridging or downstaging. Survival was better in the bridging group compared to the downstaging group, though this difference did not achieve statistical significance (*p*-value = 0.54).

Recurrence-free survival was 97.2% (35/36) at 1 year, 94.4% (34/36) at 3 years and 90% (18/20) at 5 years. Specifically, two patients developed HCC recurrence in the transplanted liver. One recurrence occurred at 12 months in a patient having previously shown complete radiological response before LT and complete HCC necrosis at explant pathology, with no lymphovascular invasion. The other patient presented with HCC recurrence 23 months after transplantation; this patient had complete radiological response before LT and 80% necrosis and no lymphatic invasion at histological analysis of the explanted liver, but showed infiltrated margins and evidence of vascular invasion. RFS after combined treatment was again not significantly influenced by inclusion in the transplant criteria at diagnosis (*p* = 0.33). The likelihood of recurrence after transplantation, thus, does not appear to be significantly different between the bridging and downstaging treatment groups.

**Fig. 5:**
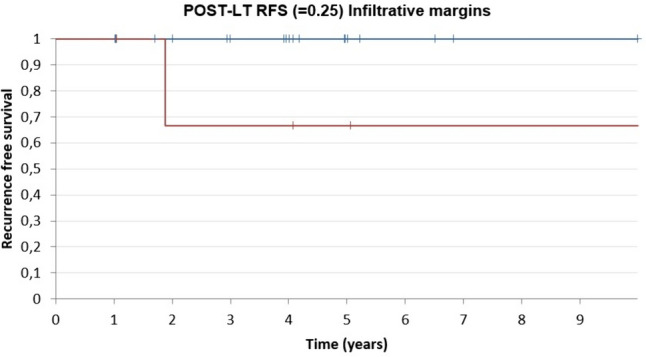
Kaplan–Meier recurrence-free survival (RFS) curve for patients with or without infiltrative margins at the explant pathology review (blue line = no infiltrative margins; red line = infiltrative margins)

In a further effort to identify possible predictors of HCC recurrence in our cohort, best subsets regression analysis was employed. The only variables that showed borderline significance were age and infiltrative margins (Table 3). Hence, these two variables were included in a multivariable model, which confirmed borderline significance only for infiltrative margins. Given the borderline significance, two Kaplan–Meier curves were calculated (*p* = 0.25) for freedom from recurrence in cases with infiltrative margins and cases without infiltrative margins (Fig. 5). No other patient-, lesion- or technique-related variable was significantly associated with HCC recurrence after LT.

**Table 3:** Best subset regression analysis of post-LT recurrence predictive factors. Only infiltrative margins (in bold) showed borderline significance in predicting post-LT HCC recurrence after the multivariate analysis

	*P* value	
	Best subset regression analysis	Multivariate analysis
*Age*	0.089	0.191
Central segment (S1, S4, S5 or S8)	0.192	
Target node diameter	0.468	
Multifocal HCC	0.502	
MW ablation	0.835	
Sequence	0.596	
Days between the two procedures	0.107	0.349
CR at first follow-up	0.225	
Radiological recurrence	0.897	
Bridging versus Downstaging	0.539	
Pre-OLT AFP level	0.513	
Necrosis (%)	0.681	
Number of mitosis/10HPF	0.251	
*Infiltrative margins*	**0.073**	**0.014**
Microvascular invasion	0.641	

*HCC* Hepatocellular carcinoma, *MW* Microwave, *CR* Complete response according to mRECIST criteria, *LT* Liver transplant, *AFP* Alpha fetoprotein, HPF High power field

### Comparison with thermal ablation only

**Fig. 6:**
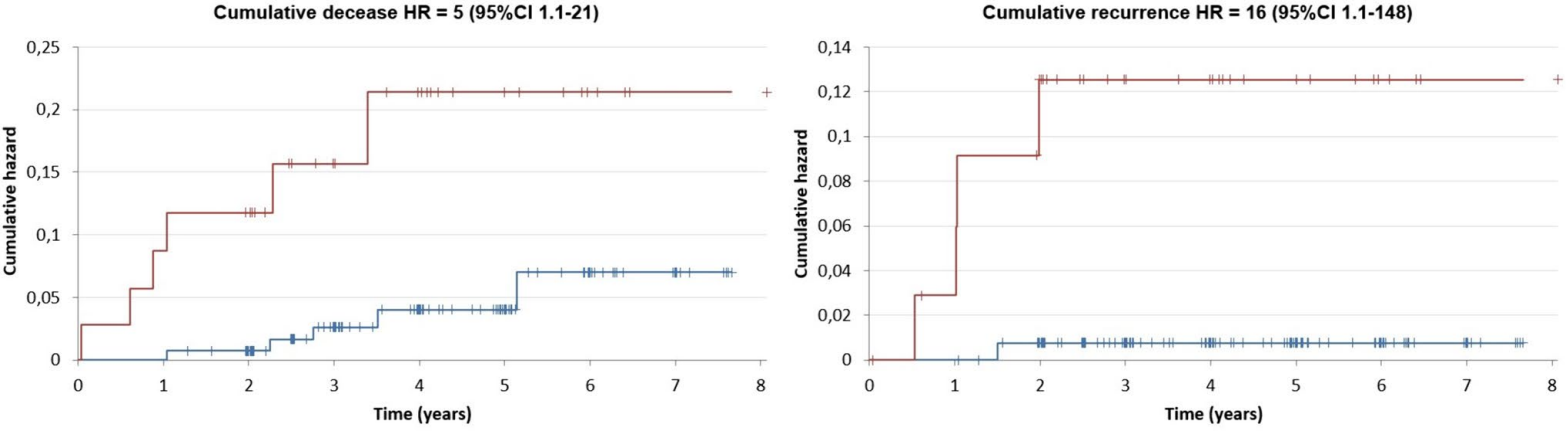
Kaplan–Meier cumulative decease and recurrence hazard ratio after LT curve for patients subjected to exclusive ablation as bridging or downstaging before LT (blue line = bridging; red line = downstaging). HR hazard ratio; CI confidence interval

A cohort of 170 consecutive patients (males, *n* = 143; females, *n* = 27) who received thermal ablation only, either by MW or RF, was selected as a comparator group. In this group, 108 (63.5%) had unifocal HCC at diagnosis, while 62 (36.5%) had multifocal HCC. Median follow-up after LT was 36.2 months. Overall and recurrence-free survival at 1, 3 and 5 years was 98.2%, 94% and 86.6%, and 99.4%, 96.2%, and 93.8%, respectively. Differently from what observed in the TA-TACE group, patients treated with TA alone in the setting of downstaging showed significantly lower overall (*p* < 0.004; Fig. 6) and recurrence-free survival (*p* < 0.001) after LT as compared to bridging patients. The cumulative death hazard ratio in patients undergoing TA only for downstaging revealed a fivefold (CI 1.1–21) higher risk of post-transplant death and a 16-fold (CI 1.1–148) higher risk of post-transplant recurrence, compared to the bridging setting (Fig. 6). The event rate per year, which is the ratio of "death" events in the two groups for each year, was comparable between the study and the control group (RR = 0.9; *p* = 0.90).

## Discussion

The primary endpoint of the study was to evaluate the percentage of necrosis achieved through combined therapy, assessed on the explanted livers. Complete necrosis of the target node was observed in 36.1% of cases examined during the explant pathology examination, with a median necrosis rate of 90%. The excellent overall and recurrence-free survival after LT indicates that combined TA-TACE serves as an effective downstaging or bridging to LT strategy in patients with HCC. Specifically, the overall survival rates at 1, 3 and 5 years were 100%, 100% and 93.3%, respectively. Considering the acceptable survival threshold proposed by the Metroticket 2.0 criteria, which is a 5 years post-transplant HCC-related death rate of < 30%, it can be concluded that the overall survival at 5 years post-transplant in our cohort was satisfactory. These findings align with a recent time-to-event meta-analysis, which indicated that combined RF + TACE therapy was associated with improved overall survival and recurrence-free time compared to TACE and RF alone in HCC larger than 3 cm [18].

The degree of necrosis observed in our series is higher compared to the findings by Wong et al. [19]. In their 2004 study, authors analyzed the percentage of necrosis in explanted livers from patients who underwent different locoregional treatments (RF, PEI or TACE) as bridging or downstaging before LT, showing complete absence of necrosis in 13/44 nodules (29.5%) versus 2/36 (5.6%) of the present study. The superior results in our series suggest a higher efficacy of the combined therapy, notwithstanding that technical advancements and the availability of better technologies for locoregional treatments over the years represent a potential confounding factor. A study by Vasnani et al. [20] compared combined therapy with RF or MW following DEB-TACE and showed a complete necrosis rate of 45% in the RF + TACE group and 53% in the MW + TACE group (*p* = 0.74). The same study also evaluated the mean necrosis percentage, which was 88.9% in the RF + TACE group and 90.5% in the MW + TACE group (p = 0.82). However, the majority of the lesions included in this study were < 3 cm in size. It is worth noting that, in our series, the RF + TACE-treated group exhibited a 22% rate of complete histopathological responses, whereas the MW + TACE-treated group had a 50% rate (*p* = 0.16). Mean necrosis percentage in the RF + TACE group was 65.9%, compared to 83.3% in the MW + TACE group (*p* = 0.099). Although both MW and RF were effective in inducing coagulative necrosis and despite the lack of statistical significance, these findings may indicate a superiority of MW over RF ablation, which should be investigated in larger studies.

In our selected patient group, no statistically significant factors were identified in predicting the risk of post-LT recurrence. However, there was borderline significance for microvascular invasion in the pathological evaluation. Literature also identifies tumor characteristics as prognostic for RFS, including high alpha-fetoprotein (AFP) levels, satellite nodules and insufficient resection margins [21]. Regarding post-LT survival, the literature has shown that factors such as patient's age, performance status, MELD score, AFP levels, microvascular invasion, satellite nodules (more commonly found in LI-RADS 5 grade HCC on imaging) and inadequate resection have an impact on OS [21]. The importance of patient age, modified Edmondson-Steiner grade, microvascular invasion and satellite nodules has also been confirmed by a 2017 study in patients undergoing liver resection for HCC [22]. The likely reason for not achieving statistical significance for any analyzed variable in our study may be attributed to the relatively small sample size.

In the present study, the rate of complete and partial radiological response was 80.6% and 19.4%, resulting in an overall 100% response rate. However, in 51.6% of cases, complete radiological response was not confirmed at histological examination. Conversely, in 6.5% of patients showing partial radiological response at first follow-up imaging, pathological analysis demonstrated complete necrosis of the target lesion. Similar to our study, Cucchetti et al. showed that 41.3% of patients undergoing neoadjuvant locoregional procedures achieved radiological CR, yet this was confirmed at histopathological evaluation in only 46.5% of cases [23]. In patients with radiological progression of disease, however, 10% showed no residual vital tumor in the pathological analysis of the explanted liver. Also the study by Vasnani et al. revealed poor correlation between radiological and pathological response and showed a higher percentage of necrosis at pathological analysis [20]. In our study, the concordance between complete pathological response and complete radiological response had an index of agreement of 58%, with a Cohen's kappa coefficient of 0.59 (CI 0.31–0.88). This concordance is slightly lower than that reported by Bargellini et al., which found an inter-observer agreement coefficient of 0.81 and an agreement between mRECIST assessment and pathological evaluation in 67.4% of cases, with radiological response being overestimated in 21.9% of cases and underestimated in 10.7% [24]. Despite this well-known discrepancy between radiological and pathological response, it is important to note that the incidence of post-LT HCC recurrence is strongly associated with the degree of radiological response assessed by mRECIST criteria [25]. In our study, the only factor positively associated with a complete radiological response with borderline significance was MW ablation (*p*-value = 0.09). This finding is in line with those by Sheta et al., who demonstrated a higher rate of radiological CR after MW + TACE compared to RF + TACE [26]. This difference might be due to MW's ability to achieve higher temperatures than RF and being less affected by the "heat-sink effect," resulting in a more extensive necrosis area in less time.

Searching for potential negative predictors of post-LT recurrence in our study revealed a trend of worsening outcomes with increasing days between the two procedures (thermoablation and TACE) using best subset regression. To our knowledge, there are currently no articles in the literature analyzing the correlation between the days elapsed between the two procedures of combined therapy and the risk of post-LT recurrence. The only variable with borderline significance in univariate analysis was microvascular invasion. Cucchetti et al. demonstrated that progression on the transplant waiting list correlates with more aggressive tumor biology and subsequently increased rates of post-LT recurrence [23]. Similarly, the study by Lee et al. [25] showed that non-response to locoregional treatments and disease progression are associated with a higher risk of post-transplant recurrence. This might confirm the role of tumor characteristics, including microvascular invasion, as a risk factor for post-LT recurrence. In our study, the time order of the two treatments did not prove to be a significant predictor of post-LT recurrence. This seems to reinforce the lack of consensus in the literature regarding protocols for combined therapy. In theory, there are good reasons to prefer either approach. Some articles suggest that TACE can reduce the cooling of the target lesion by preventing vascular flow, so performing TACE before TA could minimize the "heat-sink effect" [26–28]. On the other hand, some studies indicate that the effects of TACE could reduce lesion visibility in ultrasound, making subsequent TA more challenging [29]. Certain studies argue that performing thermal ablation as the first treatment might sensitize tumor cells to chemotherapy due to the heat effect, maximizing the effectiveness of TACE and allowing a reduced chemotherapy dose. Heat also appears to increase vascular permeability, enhancing the concentration of chemotherapeutic drug in the target lesion [26, 30].

It is worth noting that, in this study, the annual mortality rate in the study group did not significantly exceed the one of the group consisting of 170 patients treated solely with thermal ablation for nodules smaller than 3 cm (RR = 0.9, *p*-value = 0.90). This finding reinforces the significant role of combined therapy in broadening the pool of liver transplant-eligible HCC patients, without adversely affecting long-term outcomes. Moreover, patients treated exclusively with ablation for downstaging demonstrated lower post-transplant overall survival (HR = 5, 95 CI 1.1–21) and recurrence-free survival (HR = 16 CI 1.1–148) compared to patients who underwent TA as a bridging therapy. This finding aligns with a previous study by Grasso et al. [31], which investigated potential predictors of post-LT recurrence in HCC patients treated with various locoregional methods (RF, MW, TACE, PEI, etc.). That study found statistical significance associated with the size of the largest nodule, as measured on imaging, irrespective of the number and size of smaller nodules. Specifically, HCC patients with the largest nodule measuring > 3.5 cm experienced inferior 1, 3 and 5-years OS compared to those with the largest nodule diameter < 3.5 cm. Conversely, in the present study, no differences in outcomes were observed for patients treated with combined therapy based on their inclusion or exclusion from transplant criteria at diagnosis. The fact that the efficacy of neoadjuvant combined therapy did not significantly differ in the context of bridging or downstaging aligns with the observation that nodules > 3 cm have a higher recurrence rate when subjected to thermal ablation alone, likely due to the persistence of residual satellite tumor cells, which could be eliminated through the combination with TACE [12]. In keeping with this, Morimoto et al. emphasized that in patients with intermediate-sized HCC, RF + TACE treatment is more effective than RF alone, as it extends the ablation area and reduces local progression (as assessed by imaging). Furthermore, other meta-analyses have demonstrated that the combination of RF and cTACE significantly improves 1- and 3-years overall survival compared to RF alone for nodules ranging between 3 and 5 cm in diameter [32, 33].

The present study has several limitations, mainly related to the retrospective, single-center design. This contributed to the relatively low number of patients in the combined therapy group, despite the wide timeframe considered. The average follow-up duration of about 3 years was influenced by the fact that the majority of patients undergoing combined therapy were transplanted between 2020 and 2021. Inherent limitations of the techniques used include the variability of ablative techniques, sequence and the number of days between procedures. In the assessment of radiological response, another limitation lies in the sample size, as 5 patients underwent transplantation without undergoing post-procedural radiological follow-up. Radiological follow-ups were mainly performed with four-phase CT and only occasionally with MRI, which could have influenced the imaging's ability to evaluate response to combined treatment. Furthermore, the low number of deaths and post-transplant recurrences in the study sample also limits the statistical power (approximately 5%). To achieve a power of > 80%, however, 6600 patients undergoing combined therapy would be needed.

## Conclusions

In conclusion, this study demonstrates that the combination of thermal ablation and chemoembolization represents a good therapeutic option for HCC larger than 3 cm as bridging or downstaging to LT, achieving excellent overall and recurrence-free survival. With the limitations of the small sample size, no differences in terms of OS and RFS were detected comparing patients undergoing a combined approach in the setting of bridging or downstaging. Furthermore, survival outcomes were comparable to those of patients with HCC smaller than 3 cm undergoing exclusive thermal ablation. Further studies should investigate the potential advantages of MW over RF in the setting of a combined approach.
